# The effect of an early childhood obesity intervention on father’s obesity risk behaviors: the Melbourne InFANT Program

**DOI:** 10.1186/1479-5868-11-18

**Published:** 2014-02-14

**Authors:** Adam D Walsh, Sandrine Lioret, Adrian J Cameron, Kylie D Hesketh, Sarah A McNaughton, David Crawford, Karen J Campbell

**Affiliations:** 1 Centre for Physical Activity and Nutrition Research, School of Exercise and Nutrition Sciences, Deakin University, 221 Burwood Hwy, Burwood, VIC 3125, Australia

**Keywords:** Fathers, Dietary patterns, Physical activity, Sedentary behaviour, Childhood

## Abstract

**Background:**

To investigate the effect of an early childhood obesity prevention intervention, incorporating a parent modelling component, on fathers’ obesity risk-related behaviours.

**Methods:**

Cluster randomized-controlled trial in the setting of pre-existing first-time parents groups organised by Maternal and Child Health Nurses in Victoria, Australia. Participants were 460 first-time fathers mean age = 34.2 (s.d.4.90) years. Dietary pattern scores of fathers were derived using principal component analysis, total physical activity and total television viewing time were assessed at baseline (infant aged three to four months) and after 15 months.

**Results:**

No significant beneficial intervention effect was observed on fathers’ dietary pattern scores, total physical activity or total television viewing time.

**Conclusion:**

Despite a strong focus on parent modelling (targeting parents own diet, physical activity and television viewing behaviours), and beneficial impact on mothers’ obesity risk behaviours, this intervention, with mothers as the point of contact, had no effect on fathers’ obesity risk-related behaviours. Based on the established links between children’s obesity risk-related behaviors and that of their fathers, a need exists for research testing the effectiveness of interventions with a stronger engagement of fathers.

## Background

The worldwide prevalence of childhood obesity is of concern. In Australia, 21 to 24% of children aged two to sixteen years are overweight or obese [1]. These data are comparable with those from other developed nations [2-4]. Childhood obesity is an acknowledged risk factor for the development of non-communicable diseases [5] and whilst the determinants of childhood adiposity are multifaceted, energy-balance behaviours are important predictors [6,7].

Evidence suggests that weight status and its behavioural determinants track throughout childhood into the adult years [8-10]. Children’s learning about food, physical activity and sedentary behaviour is considerable during their formative years; through the observation and imitation of significant others [11-15] and these foundation years are likely to be where the influences on children’s behaviours are greatest [16-18]. The influence of parents on children’s eating and activity is considered pivotal, although most research focuses on maternal influence.

Evidence linking fathers’ and children’s dietary, physical activity and sedentary behaviours is beginning to accumulate [19-22]. For example, Moore and colleagues reported that young children (4-7 years) were 3.5 times more likely to be active when their father was also active, with the likely mechanisms being role modelling, sharing of activities and support for physical activity [19]. This is supported by work from Cleland et al. who reported that paternal reinforcement and direct support for physical activity was positively associated with 4-7 and 10-12 year old boys’ physical activity [22]. Associations between fathers’ and children’s dietary intake and physical activity have also been examined by Morgan and colleagues [23]. They reported that fathers participating in a healthy lifestyle program targeting obesity risk behaviours achieved significant weight loss and improved health-related outcomes, whilst their co-participating children improved their eating and physical activity behaviours [23]. Whilst scarce, research focusing solely on dietary behaviours reveals similar results. For example, Hall et al. and McIntosh et al. both reported positive associations between fathers’ eating habits and that of their children [21,24]. Collectively, these studies provide strong support for the notion that fathers’ eating and physical activity behaviours influence their child’s health behaviours.

In addition to the associations between children’s and fathers’ health behaviours, mothers’ and fathers’ health behaviours have also been shown to correlate. For example, Feunekes et al. and Oliveria et al. both reported correlations between mothers’ and fathers’ macro and micro nutrient intakes [25,26] whilst Northstone et al. and Lioret et al. reported correlations between identified dietary patterns in mothers and fathers [27,28]. These relationships suggest that the dietary behaviours of one parent may influence the dietary behaviours of the other [29]. It was in the context of the associations between the diets of mothers and fathers and the diets of mothers and their children, that the Melbourne InFANT Program was developed. The InFANT program is an early childhood obesity prevention intervention with a strong focus on parent modelling using mothers as the point of contact, the aim of which was to test the effectiveness of an early childhood obesity prevention intervention, focussing on parenting skills which support the development of positive diet and physical activity behaviours, and reduce sedentary behaviours in infants from three to 18 months of age. Results of the study previously reported have included the effects of the intervention on children’s and mother’s diet and physical activity [30], the correlation between mothers’ and fathers’ diets [28]; an analysis of the extent to which the association between maternal education and infant diets is mediated by mother’s diets [31]; and an analysis of the effect of the intervention according to maternal education and age [32].

The aim of the present study is to test the hypothesis that this early childhood obesity prevention intervention would improve the dietary, physical activity and sedentary behaviours of fathers via the behaviour and role modelling of mothers, noting that mothers have traditionally been considered the gatekeepers of the family food environment [33,34].

## Methods

### Study design

The Melbourne Infant Feeding Activity and Nutrition Trial (InFANT) Program is a cluster-randomized controlled trial (RCT) undertaken within pre-existing first-time parent groups (parent support groups for first-time parents of recently born children), which has been described in detail elsewhere [30,35]. Briefly, the intervention, delivered by a dietitian comprised of six sessions delivered at three month intervals (15 months total) during the regular meeting time of the first-time parents' group. The intervention incorporated a range of modes of delivery (e.g. brief didactic sessions, take-home DVD and newsletters) and educational strategies and covered topics such as basic nutrition principles, parental modelling of eating, physical activity and sedentary behaviours, limiting sedentary behaviour and provision of opportunities for physical activity. Mothers were asked to share and discuss the provided resources to fathers and other carers of their child. The control group families received usual care from their MCH nurse.

First-time parent groups were selected across areas of all socio-economic position using a two-stage random sampling design. Sixty-two first-time parent groups were randomly selected from 14 local government areas within a 60 km radius of the research centre (Deakin University in Burwood, Victoria, Australia). Groups that consented to participate were randomly allocated to intervention or control arms. Inclusion criteria were English literacy and a minimum of eight parents in the groups consenting to participate (or six parents in low socio-economic position area groups).

**Figure 1:**
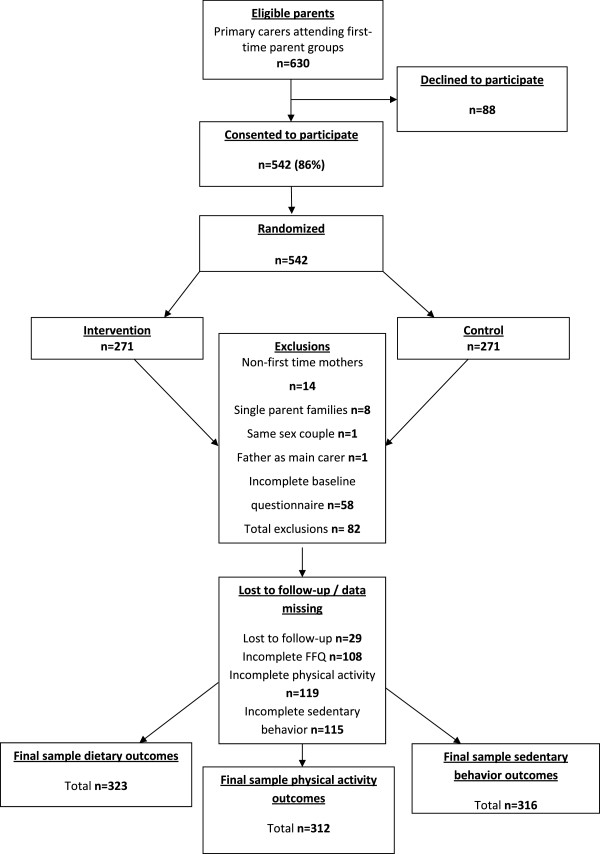
Participant response, exclusions and follow-up.

Exclusion criteria for this study included non-first time parents (n = 14), single parent families (n = 8), father as main carer (n = 1), same sex couple (n = 1) and 58 fathers who did not complete the baseline questionnaire. The sample available at baseline and described in Table 1 consisted of 460 fathers. Post-intervention, 29 families were lost to follow-up and data were missing for 108 -119 fathers depending on the outcome (i.e. diet, physical activity or sedentary behaviour). To minimize the effect of incomplete or missing data, analyses were conducted on separate samples for each main outcome, leading to a different sample for each (Figure 1): 1) dietary outcomes (n = 323), 2) physical activity (n = 312), and 3) sedentary behaviour (n = 316).

**Table 1:** Baseline characteristics of fathers randomized to the intervention and control groups

	**Intervention**	**Control**	**All**
	**n = 224**	**n = 236**	**n = 460**
Age (years)	33.9 (4.8)	34.5 (5.0)	34.2 (4.9)
BMI (kg/m^2^)	28.0 (5.1)	27.6 (5.1)	27.8 (5.1)
*BMI Category* (%)			
Healthy weight	28.6	33.0	30.8
Overweight	46.3	45.5	45.9
Obese	25.1	21.4	23.3
*Education level,* % (95% CI)			
Low	26.1 (18.4; 33.8)	27.4 (21.2; 33.5)	26.7 (21.8; 31.7)
Intermediate	33.0 (26.5; 39.6)	33.0 (27.3; 38.8)	33.0 (28.7; 37.4)
High	40.9 (31.2; 50.6)	39.6 (30.3; 48.9)	40.2 (33.5; 46.9)
Country of birth, % (95% CI)			
Australia	75.1 (70.1; 80.1)	79.6 (74.4; 84.8)	77.3 (73.7; 81.0)
Other	24.9 (19.9; 29.9)	20.4 (15.2; 25.6)	22.7 (19.0; 26.3)
Language spoken at home, % (95% CI)			
English	95.2 (91.8; 98.6)	96.1 (93.3; 98.9)	95.7 (93.4; 97.9)
Other	4.8 (1.4; 8.2)	3.9 (1.1; 6.7)	4.3 (2.1; 6.6)

Figures are means (SD) unless stated otherwise. CI = Confidence Interval.

Process evaluation was undertaken at each session and sought participants’ feedback on the usefulness and relevance of the program. Respondents were asked to rate on a 4-point scale from “not at all useful/relevant” to “very useful/relevant.” If participants did not attend sessions, data collection occurred in the home. Intervention materials were sent to non-attending parents, and a researcher telephoned to invite questions. Mothers were asked to share and discuss the provided resources to fathers and other carers of their child although no formal assessment of whether these resources were shared or discussed with other carers occurred.

The Melbourne InFANT program was approved by the Deakin University Human Research Ethics Committee and the Victorian Government Department of Human Services, Office for Children, Research Coordinating Committee. Informed written consent was obtained from all participants.

### Measurements

Data were collected using self-administered questionnaires provided to fathers (via mothers) at baseline and post-intervention and related to diet, physical activity and television viewing behaviours. Questionnaires were returned at the next session attended or by mail and checked by research staff. Demographic and socio-economic variables included fathers’ age; marital status; country of birth; main language spoken at home; employment status and education level. Weight and height were also self-reported with body mass index (BMI) calculated as weight (kg)/height (m^2^).

Fathers’ (and mothers’) dietary data was collected at baseline and post-intervention using the previously validated [36] Cancer Council Victoria Food Frequency Questionnaire (FFQ). This FFQ is an updated version of the semi-quantitative FFQ specifically developed for the Melbourne Collaborative Cohort Study [37] and has been previously validated using seven-day food diaries where correlation coefficients for energy-adjusted nutrient intakes ranged from 0.28 (vitamin A) to 0.78 (carbohydrate) [36]. The FFQ has 10 response options for 98 food items ranging from “never” to “three or more times per day”. Fathers were asked to indicate how often they had consumed each food or beverage item over the preceding 12 months. These data were converted into daily equivalent frequencies according the Cancer Council Victoria protocol. Also included in the FFQ were 11 additional questions relating to the type and amount of milk consumed (glasses per day); the amount of diet and non-diet soft drinks consumed (glasses per day); the type and amount of bread consumed (slices per day); the number of eggs per week; and the frequency of consumption per week of both alcoholic and hot beverages.

Fathers’ total physical activity (both leisure and occupational) was assessed at baseline and post-intervention using the previously validated Active Australia Survey [38]. Fathers were asked to estimate the total duration they spent walking continuously (for at least 10 minutes); and doing both vigorous and moderate physical activity the week preceding the interview. Total physical activity time (in min/week) was calculated by summing the time spent in walking and moderate activity and twice the time spent in vigorous activity in accordance with The Active Australia survey protocol [39]. Any times greater than 840 min/week for a single activity type were truncated at 840 min/week (14 h). In addition, total time in all activities that were greater than 1680 min/week (28 h) were truncated at 1680 min/week.

Fathers also reported the usual time spent watching television or videos/DVDs on weekdays and weekend days at baseline and post-intervention using an abridged version of a previously validated questionnaire [40]. An average daily time (in min/d) was calculated and weighted from the values reported for each type of day (weekday *vs.* weekend day). Watching television or videos/DVDs is used as a proxy of sedentary behaviour. Reported durations and total television viewing time were truncated at 1080 min/day (18 h).

### Statistical analyses

Based on similarities in food type, energy density and context of consumption, the 98 assessed foods and beverages were condensed into 54 groups. Frequencies of consumption of foods within each group were summed. Dietary patterns were derived in fathers at baseline in a previous study [28] using principal component analysis (PCA) with varimax rotation for the fifty-four food groups [41] accounting for 23.5% of the explained variance. The number of patterns was selected considering eigenvalues >1 · 0, the scree plot and the interpretability of the patterns [42]. To interpret the results and calculate the scores, we considered the items most strongly related to the pattern, i.e. those for which the loading coefficient was > |0 · 15|. This threshold was chosen accounting for the overall range of loadings observed in our data (i.e. the ranking of foods in the pattern) and both the interpretability and differentiation of each pattern as well as previous studies of dietary patterns [43,44] resulting in the inclusion of 41 of the 54 food groups (Table 2). Due to the longitudinal design of our analyses, the factor scores for each dietary pattern identified at baseline were calculated at the individual level at both baseline and post-intervention. This was performed by summing the observed standardized frequencies of consumption per food group at each point in time weighted according to the factor loadings estimated at baseline. These factor loadings are either positive or negative and resulting scores for a given dietary pattern are the linear combinations of the initial variables (standardized frequencies of consumption per food group) weighted according to the factor loadings. A negative score indicates food groups that they are inversely correlated to a dietary pattern while a positive score indicates positive correlation to a dietary pattern.

**Table 2:** **Food groups loading**^**1**^ **> 0.15 on each of the dietary patterns identified in fathers at baseline**[28]

**Dietary pattern**	**Food groups (loading)**
Pattern 1 Fruits	Fruits (common fresh: 0.27, other fresh: 0.29, tinned: 0.25), whole meal crackers (0.33), confectionary (0.33), non-fried fish (0.21), ricotta and cottage cheese (0.20), salty and non-whole meal biscuits (0.20), breakfast cereal (0.18), nuts other than peanuts (0.18), yoghurt (0.17), cakes and pastries (0.17), sweet biscuits (0.15), margarine or oil on vegetables (0.17), oil and vinegar salad dressing (0.16)
Pattern 2 Potatoes and vegetables	Cooked vegetables (0.48), raw vegetables (0.45), legumes (0.42), potatoes cooked with no added fat (0.29) potatoes cooked with fat added (0.24), alcohol (0.16), cakes and pastries (-0.16)
Pattern 3 High-fat foods	Fried fish (0.35), sausages (0.34), red meat (0.29), pizza (0.27), white bread (0.27), vegetables with butter added (0.20), savory pastries (0.17), salad dressing (0.17), potatoes cooked with fat added (0.16), bread other than white (-0.23), breakfast cereals (-0.23), yoghurt (-0.19), reduced fat milk (-0.18)
Pattern 4 High energy snack and processed foods	Potato chips (0.28), chocolate products (0.28), ketchup (0.28), delicatessen meats (0.26), sweet biscuits (0.23), ice-cream (0.23), cheese (0.22), potatoes cooked with fat added (0.21),, cakes and pastries (0.20), savory pastries (0.20), potatoes cooked with no added fat (0.20), peanut products (0.18), bread other than white (0.17), yeast extract/spread (0.17), diet carbonated soft drinks (0.16), oil and vinegar salad dressing (-0.18)

^1^Absolute values.

Factor loadings for food group indicated in parentheses.

The primary analytic approach was based on the intention-to-treat principle, with all participants completing follow-up surveys included in the treatment arm to which they were assigned. Baseline comparability of intervention and control arms was assessed using Chi square tests (categorical variables) and linear regression analyses (continuous variables). Analysis of covariance (ANCOVA) was used to measure the impact of the intervention on each of the six outcomes of interest (the four dietary patterns scores, total physical activity and TV viewing time). Resulting residuals were checked in terms of distribution and independence, and dependent variables consequently transformed when needed (square-root transformations for physical activity and TV viewing time). Additional analyses of covariance were undertaken which also accounted for three well understood covariates of the outcomes studied; educational level, age, and weight status defined in 2 categories (namely BMI < 25 kg/m2 and BMI ≥ 25 kg/m2). Clustering by first-time parents group was accounted for in all models. Adjusted parameter estimates and 95% CI were calculated. The accepted significance level was set at 5%. Analyses were computed using Stata software (release 12; StataCorp LP, College Station, TX, USA).

## Results

Table 1 presents the baseline characteristics of participating fathers. There were no baseline differences between those randomized to the control and intervention groups. Fathers’ mean age was 34.2 years (s.d.4.9) and mean BMI was 27.8 kg/m^2^ (s.d.5.1). The majority (40.2%) of fathers had achieved a university education, 33.0% trade and certificate qualifications and 26.7% secondary schooling or below. English was the main language spoken at home with slightly over three quarters of the sample born in Australia. There were no significant differences in baseline characteristics between those lost to follow-up or excluded from analyses and those retained at 15 months.

Total physical activity (min/week) was found to be sufficient for health in approximately 73% of participants irrespective of treatment group according to Australian National Guidelines [45]. Participant screen time use was approximately 2.5 hours per day. This is comparable to previously reported results in the adult population. The mean change in dietary pattern scores, screen time and total physical activity in both groups at baseline and follow-up is presented in Table 3. There was no beneficial intervention *vs.* control effect in dietary pattern score, screen time or total physical activity at either time point when adjusted for baseline score or when further adjusted for paternal education, paternal age or paternal BMI category (BMI < 25 kg/m^2^ and BMI ≥ 25 kg/m^2^).

**Table 3:** Adjusted fathers’ dietary patterns scores, physical activity and sedentary behaviors at post-intervention

	**Baseline**				**Follow-up**		**Difference (intervention - control), adjusted for baseline (95% CI)**^**2**^	**P-value**	**Difference (intervention - control), adjusted for baseline and covariates (95% CI)**^**3**^	**P-value**
	**Intervention**		**Control**		**Intervention**	**Control**				
	**n**	**Mean (SD)**	**n**	**Mean (SD)**	**Mean (SD)**	**Mean (SD)**				
"Fruits" pattern scores	163	-0.10 (1.45)	160	0.11 (1.61)	-0.16 (1.17)	0.16 (1.46)	-0.21 (-0.44;0.02)	0.076	-0.25 (-0.48; -0.02)	0.033
"Potatoes and vegetables" pattern scores	163	-0.12 (1.17)	160	0.12 (1.24)	-0.06 (1.17)	0.06 (1.26)	0.01 (-0.23;0.26)	0.89	0.02 (-0.23;0.27)	0.85
"High-fat foods" pattern scores	163	0.08 (1.64)	160	-0.08 (1.27)	0.05 (1.14)	-0.05 (1.48)	0.03 (-0.27;0.32)	0.86	0 (-0.30;0.30)	0.99
"High-energy snack and processed foods" pattern scores	163	0.08 (1.36)	160	-0.08 (1.38)	-0.04 (1.32)	0.04 (1.39)	-0.17 (-0.39;0.05)	0.13	-0.16 (-0.37;0.06)	0.15
Total physical activity (min/week)^4^	159	403.5 (373.9)	153	449.0 (434.5)	424.3 (390.9)	464.0 (396.1)	-0.58 (-2.67;1.51)	0.58	-0.92 (-2.96; 1.12)	0.37
Sedentary behaviour (min/day)^4^	160	159.5 (86.5)	156	165.0 (99.9)	157.0 (135.4)	155.6 (132.0)	0.21 (-0.76;1.17)	0.67	0.24 (-0.74; 1.22)	0.63

^2^Adjusted for baseline value; ^3^Adjusted for baseline value, paternal education level, paternal age, and paternal pre-pregnancy weight status defined in 2 categories (namely BMI < 25 kg/m^2^ and BMI ≥ 25 kg/m^2^); ^4^Square-root transformation of the dependent variable in the linear regression analysis.

## Discussion

This study sought to examine whether an early childhood obesity prevention intervention incorporating a parent behavioural modelling component, altered obesity risk behaviours of fathers. The findings show that the intervention, with mothers as the point of contact, had no beneficial impact on the diet, physical activity and sedentary behaviours of fathers. The presence of a beneficial outcome in mothers (who were the point of contact for this intervention) but not fathers, supports the notion that direct contact in an intervention may be necessary to change fathers’ health behaviours.

Reasons for the absence of any impact on fathers’ obesity-related behaviours may have included paternal attitudes towards infant diet and physical activity behaviours (i.e. they may perceive this to be a maternal domain), that the intervention materials were not shared or discussed with them by their partners or that simply, it was construed by fathers as irrelevant to them. Qualitative work is needed to gain an understanding of fathers’ perceptions about their role in influencing children’s eating and activity behaviours. Whilst qualitative understandings of father’s perceptions of their roles in the first year of life exist [46-48], qualitative understandings of mother’s perceptions of father’s roles in these domains would also be valuable.

The present study identified four dietary patterns in fathers. Common characteristics were identified between the present findings and a study involving Australian women aged 25–30 and 50–55 years [49]. That study used comparable methodology but included dietary patterns with a higher level of detail regarding food items as a higher number of food groups were considered in that study. Two identified patterns labelled ‘Processed meat, meat and takeaway’ and ‘High-fat and sugar foods’, were similar to patterns we identified (namely ‘High-fat foods’ and ‘High-energy snack and processed foods’).

International studies have also shown some consistency with these findings. Crozier et al, identified two main patterns in their UK study involving 6125 non-pregnant women aged 20–34 years [43]; ‘Prudent’ and ‘High-energy’. The patterns identified in the present study, ‘Fruits’ and ‘Potatoes and vegetables’ have similar characteristics to the ‘Prudent’ pattern. Conversely, patterns with high loadings for red and processed meats, refined grains, and processed foods have often been labelled as ‘Western’ [44], similar to the ‘High-fat foods’ and ‘High-energy snack and processed foods’ patterns identified in the present study.

Literature suggests an important role of fathers in children’s development; however scant evidence exists regarding the influence of fathers on the obesity risk-related behaviours of very young children [50]. Maternal associations with young children’s diet and physical activity behaviours through role modelling and other practices has been widely reported [11,15,16,51], whereas reports on the paternal associations with these behaviours has occurred with less frequency and generally only in school-aged children or adolescents [26]. In one of the few studies to examine the associations between child behaviours and both parents, Moore et al, in their cross-sectional analysis of data from the Framingham Children’s Study, described associations between children and both parents. They reported that children of active mothers were twice as likely to be active as children of inactive mothers; whilst having an active father was associated with children who were 3.5 times more active [19]. Based on these results, it may be that stronger intervention effects could be achieved if interventions are delivered to both parents.

The findings in the present study provide important insights into first-time fathers’ dietary, physical activity and sedentary behaviours and highlight that overweight and obesity is highly prevalent in this group. Whilst the obesity risk behaviours of men and fathers in general have been reported previously [2,52,53], the subgroup of first-time fathers has not, to our knowledge, been studied. The diet, physical activity and sedentary behaviour profiles of first-time fathers described in this study are an important snap-shot of the behaviours first-time fathers display to their children. Greater knowledge of these behaviours will allow the design of appropriately targeted, family-based interventions concerning the health behaviours of infants and young children. Family-based interventions targeting both parents, that influence the development of healthy family physical activity and food environments for infants and young children, may prove to be important in addressing childhood obesity levels.

Our investigation was novel in its focus on first-time fathers. Study strengths included a randomized design, high response and retention rates and the large percentage of fathers that completed data collection despite their partners being the point of contact. There were some limitations to the study that should be noted. Factor analysis inherently involves some subjectivity, however, a review by Newby and colleagues reported considerable reproducibility of dietary patterns across most studies in adults [44]. Furthermore, since identifying the effects of the intervention was the goal of this study, the subjective nature of dietary pattern identification is unlikely to have affected our findings. The ability of the FFQ to detect change is unclear. The FFQ has been used to previously to assess dietary change in large intervention studies [52] and whilst it has been validated for dietary information collection, there is potential uncertainty around measuring dietary change.

Dietary, physical activity and sedentary behaviour data was self-reported or possibly reported by mothers on behalf of fathers and therefore susceptible to social desirability bias and imprecision, however the tools used for collection of these data have been previously validated [36,38]. As discussed in the methods, we have no measure of the extent to which the intervention was delivered to fathers by their partners. This may have impacted the findings seen here.

## Conclusion

The Melbourne InFANT program was an early childhood obesity prevention intervention with a strong focus on parent modelling, promoting the development of healthy eating and active play behaviours from early infancy. The primary point of contact for the intervention was mothers, and we have shown here that the intervention had no beneficial effect on fathers’ health behaviours. Based on the established links between children’s obesity-related behaviours and those of their fathers, a need exists for investigations testing the effectiveness of interventions delivered directly to fathers (or both parents) in the context of the dietary and physical activity environments of infants and young children. These investigations could also examine the attitudes of fathers towards children’s diet and physical activity behaviours to allow a stronger understanding of why fathers do or do not engage in family-based interventions as well as why fathers do or do not engage in healthful or un-healthful behaviours for themselves and their children. Accordingly, assessment of the impact of obesity prevention interventions delivered to both parents would allow the quantification of the additional effects of including fathers in such programs.

## Abbreviations

BMI: Body mass index; FFQ: Food frequency questionnaire; InFANT: Infant feeding activity and nutrition trial; RCT: Randomized controlled trial.

## Competing interests

The authors declare no conflicts of interests.

## Authors’ contributions

AW drafted and edited the manuscript, contributed to the interpretation of the results and had primary responsibility for the final content. SL conducted the statistical analysis, contributed to the interpretation of the results and drafted and edited the manuscript. SAM conducted the dietary data collection, guided the statistical analysis, contributed to the interpretation of the results and edited the manuscript. DC guided the statistical analysis, contributed to the interpretation of the results, drafted and edited the manuscript. AJC contributed to the interpretation of the results and drafted and edited the manuscript. KH designed and led the Melbourne InFANT Program, guided the statistical analysis, contributed to the interpretation of the results, drafted and edited the manuscript. KJC was the principal investigator on the Melbourne InFANT Program. She designed and led that study, conducted the dietary data collection, guided the statistical analysis, contributed to the interpretation of the results and drafted and edited the manuscript. All authors read and approved the final manuscript.
